# I am just a sister: a field study on sister as mother role from Guangdong, China

**DOI:** 10.3389/fpsyg.2024.1361096

**Published:** 2024-09-23

**Authors:** Zhuowen Feng, Yunying Ye, Haojing Wang, Jiuling Wang

**Affiliations:** ^1^College of Literature and News Communication, Guangdong Ocean University, Zhanjiang, Guangdong, China; ^2^School of Civil and Commercial Law, Beijing Institute of Technology, Zhuhai, Guangdong, China

**Keywords:** motherhood, sister-motherhood, family structure, women's roles, in-depth interview

## Abstract

This study aimed to explore *sister-as-mother* (*jieximuzhi*) roles in families after the implementation of China's *universal two-child* (*quanmianerhai*) and *three-child policies* (*saihaizhengce*). By conducting in-depth interviews with 10 sisters from Guangdong Province who fit the sister-motherhood profile, this study investigated their motivations for taking on maternal duties, the characteristics of sister-motherhood roles, and the impact of these roles on the sisters. The results revealed that in families with two or more children, sisters passively assume mother-like responsibilities primarily due to biological age differences and parental neglect, as well as their personal initiative to a certain extent. Additionally, the impact of sister-motherhood roles on sisters includes aspects such as time pressure, financial strain, and increased family intimacy. This study elucidated the phenomenon of sister-motherhood roles that emerge with changes in family structure in China while recognizing the diversity of women's roles within the family, appreciating the challenges faced by sisters in sister-motherhood roles, and providing insights into family labor division and education models.

## 1 Introduction

In 1971, China implemented its family planning policy to curb the nation's excessive population growth. This policy led to a unique family structure, which predominantly consisted of four grandparents, two parents, and a single child. This was especially noticeable when the first generation of only children began forming families (Mo and Bao, 2007). However, this change soon led to challenges: most notably, the increasing aging population. To foster a healthier demographic structure and ensure the balanced growth of the population, China has been continuously revising its fertility policies. The *universal two-child* (*quanmianerhai*) policy was launched on January 1, 2016, and further expanded to the *three-child policy* (*saihaizhengce*) on May 31, 2021. According to the 2021 Health Development Statistical Bulletin, China recorded 10.62 million births, with 41.4 and 14.5% being second children and coming into families with three or more children, respectively.

Since the implementation of reform and opening up, Chinese society has transformed from the planned economic system to marketization, leading to a transition in reproductive labor and child care which are shifted to family (Cook and Dong, 2011). One significant change in child care is the involvement of kinship networks, particularly with the help of grandparents taking care of children (Xiao, 2014) or older siblings assume family caregiver roles when grandparents become elderly (Wang and Long, 2019). Moreover, the child care is also significantly affected by the family planning policies. The evolution of China's *one-child* to *universal two-child* and then to *three-child policies* has led to a notable phenomenon in which many families have siblings with an age gap of over a decade. This change has given rise to a family structure often characterized by an “adolescent first-child” and an “infant second child” as well as frequently overwhelmed and exhausted parents (Song, 2021). In such families, the burden often falls on the older sibling, typically resulting in older sisters assuming roles akin to those of a second mother. These elder sisters often take on significant responsibilities, including the care and guidance of their younger siblings' daily lives and education (Wang and Long, 2019; Chen and Shi, 2017). This study focuses on the concept of sister-motherhood, and explores the initiative and passivity of elder sisters taking on maternal roles. In addition, this study delves into the characteristics of this phenomenon, the extent of sister involvement in maternal duties, and the impact of these responsibilities on their personal development and wellbeing.

Since around 2010, Chinese academics have increasingly focused on the concept of motherhood and continuously drive innovation in this field. Researchers have expanded their scope to include unique groups like “Laopiao (floating elderly)”, and focused on the challenges of “Laopiao” mothers, who migrate to their children's cities taking care of grandchildren experience anxiety as a result of their lack of social support and the emotional bond that distance erode between them and their children and grandchildren (Bu and Wei, 2020). Similarly, researchers have discovered the primary concern of floating grandparents' everyday lives is providing caregiving duties and how their hectic schedules both enhance and diminish their experience of aging. The daily schedules of migratory grandparents are structured around the requirements of their adult children and grandchildren in the private sphere. They are mainly cut off from other facets of social life in a new setting as they immerse themselves in assimilating and learning childrearing concepts and techniques, and they were too occupied to deal with anything else (Zhang, 2020). Researchers also found despite of the access to daycare centers in contemporary China, paternal grandparents from the countryside tremendously increase childcare to their son's families, particularly relieving mothers with intense workloads and high demanding schedules. Their contribution to households indicates strong family obligations still remain central role and strengthen intergenerational solidarity (Chen et al., 2011). Furthermore, researchers have argued the migration of grandparents solve the childcare problems when both parents are involved in paid employment and they are conducive to adult children's market force production and facilitate social reproduction (Qi, 2018). Besides, researchers reported the health implications of grandparents who engage in high intensity care, the results showed their health decline as the intensive caregiving load and also revealed the meanings of nurturing roles (Chen and Liu, 2012). However, a noticeable gap in the study of sister-motherhood within China persists. Therefore, this research aims to bridge this gap by integrating existing motherhood studies while adhering to academic standards, and objective principles. Employing rigorous methods and a scientific approach, the study utilizes in-depth interviews to thoroughly investigate the nuances of sisterly motherhood. This exploration is intended to enrich the understanding of family dynamics and the diversity of women's roles. By filling the gap in sister-motherhood, the research seeks to further enrich and expand related studies in motherhood and feminist research, thus potentially offering new insights and directions for future inquiries into the concept of motherhood.

## 2 Literature review and research question

### 2.1 Sister-motherhood

In recent years, “motherhood” has emerged as a vital aspect of gender studies. It encompasses women's acknowledgment and embodiment of the maternal role and entails responsibilities like caregiving and nurturing. Essentially, motherhood represents a set of activities and relationships socially constructed around the care and upbringing of children (Arendell, 2000). Traditionally, literature on child care practices have been mainly concentrated on motherhood studies, for child care has traditionally been recognized as a female responsibility. However, Jaggar (1983) broadened this concept and defined it as any relationship where one individual raises and cares for another, regardless of blood relation. Under this expanded definition, the nurturing roles played by older sisters, grandmothers, and even hired nannies can also be classified as forms of motherhood. This inclusive perspective allows for a deeper understanding of caregiving beyond traditional maternal bounds. Chodorow et al. presented a unique perspective on motherhood in contemporary society. The central inquiry explored in the book is whether or not the gender division of labor revolves around the maternal responsibilities of women why women tend to take up the obligations of motherhood (Chodorow, 2023). She posited that motherhood possesses a self-reproducing quality, wherein mothers nurture daughters who inherently develop maternal capabilities and inclinations. These abilities and desires are rooted in the formation and evolution of the mother-daughter relationship. Moreover, Chodorow argued that daughters, through identification with and emulation of their mothers, come to prioritize the interdependence of the mother-daughter bond. This dynamic fundamentally shapes their approach to motherhood in adulthood, thereby facilitating the perpetuation and reinvention of maternal roles across generations. As a result, females have a higher likelihood than boys of assuming the role of family caregivers at a young age (Mayseless et al., 2004). Similarly, Chodorow's insights are highly relevant to the Chinese setting, there remains a distinct gender disparity in chinese family care, with women of all age groups primarily responsible for childrearing and domestic tasks (Zhao, 2020). Regarding cultural notions, the traditional Chinese ethic of familism is a value system that safeguards and advances the stability of a society predicated on the family as the fundamental unit. Under this system, blood relatives share a unique, inherent, and intimate bond, and are entrusted with boundless and unconditional duties and obligations to provide support and care for each other. In a collective society that prioritizes family over individual interests, women from young ages are readily encouraged and acknowledged to take on family obligations (Li, 2019). The implementation of the three-child policy is expected to lead to a rise in family support for caretaking (Zheng and Zhang, 2021).

In English, two terms are commonly used to describe motherhood: “motherhood” and “mothering”. Although these terms are often used interchangeably in the literature, they encompass a wide array of perspectives, encompassing aspects that are static and dynamic, normative and practical, and cultural and individual (Wu, 2021). Rich distinguished these two concepts in her groundbreaking work. She posited that “motherhood” represents a static, institutional, and societal concept, essentially an “institutional motherhood” governed by patriarchal structures. This version of motherhood is molded by societal expectations and historical constructs of the maternal role and is often shaped by mainstream male culture. It can also be understood as a “maternal role” or “maternity.” On the other hand, “mothering” is conceptualized as an experiential form of motherhood, which is more dynamic and practical. It pertains to the process and experiences involved in becoming or being a mother, which is characterized by personal, subjective feelings and practices. This is sometimes referred to as “maternal nurturing” or “motherhood practice.” While these two interpretations overlap, they are not mutually exclusive. Claudia Card critiqued the concept of institutional motherhood for its oppressive and patriarchal nature. Consequently, in recent years, feminists have increasingly advocated for translating “motherhood” as “maternal empowerment” rather than maintaining its traditional connotation. This change aims to challenge oppressive structures and allow for a more liberated and emancipated understanding of maternal practice (Card, 2004; Gao, 2021).

In this study, the focus on maternal motherhood leans toward the concept of “mothering”, which emphasizes the maternal experience. Although older sisters do not undergo the birthing process, they actively participate in the upbringing of their younger siblings, thereby acquiring motherhood experiences through daily caregiving activities. Crucially, they subjectively perceive themselves as having embraced the responsibilities and roles akin to those of a mother. Simultaneously, the maternal role assumed by the elder sister is also shaped by societal and familial expectations. Hence, sister-motherhood is identified as a unique form of motherhood. The conceptualization of “sister motherhood” can be understood through three dimensions: (1) The Chinese saying “An elder sister is like a mother” reflects traditional gender roles and expectations and further emphasizes the gendered aspect of caregiving. These sisters are expected to fulfill the role of “mothers” and nurture their siblings as if they were their own offspring. During the fieldwork, we discovered that these sisters, despite their resistance to the externally imposed discourse, their identities were institutionalized as mothers and patriarchal structures dictated their mothering practices. (2) The collection of childrearing activities encompassed in this context were explicitly recognized and accepted by sisters as forms of mothering practices. As sisters, they were dedicated to the physical and emotional labor involved in nurturing their roles as mothers. (3) Crucially, it encompasses the self-identity of the sisters. Through the field investigation, we discovered that despite of their internal conflicts and passiveness, they identify themselves as the maternal roles for siblings they look after and take it for granted. The self-identification is strongly influenced by the child-care practices and the institution of patriarchy. In this context, the older sister either actively or passively steps into a maternal role within the specific dynamics of her family. While overlapping elements exist between sisterhood and motherhood, it's important to recognize the distinct aspects of each. Sister-motherhood is a distinct, multifaceted experience, which reflects the complex interplay among personal agency, familial roles, and societal expectations.

### 2.2 Intensive motherhood, extensive motherhood and alloparenting

As a result of the modernization of Chinese society and the emergence of the one-child generation in particular, parenting practices have evolved substantially. The 1980s catchphrase “little emperors” reflected the fact that children became the focal point of the family. In 1980, Parents Must Read, the first family parenting periodical in China, was published with the intention of guiding parents in the scientific wayward development of intelligent, healthy, and contemporary children. Books and media pertaining to family education began to adopt western child development psychology as their scientific foundation after the turn of the 21st century. These sources emphasized the need for a shift in family parenting from a “adult perspective” to a “child-centered” approach, while also underscoring the criticality of early education and emotional communication (Zheng and Zhang, 2021). Therefore, in contemporary China, the role of mothers transcends the confines of traditional motherhood to confront a plethora of new challenges and transformations. To navigate these complexities, mothers are often called upon to assume additional roles within the family, which necessitate a heightened focus on self-sacrifice and dedication to their children's needs. In the 1980s, Hays introduced the concept of “intensive motherhood,” a paradigm that posits the mother as the optimal caregiver for a child (Hays, 1996). This ideology expects mothers to prioritize the physical and emotional wellbeing of their children above all else and dedicate themselves tirelessly to their familial responsibilities and providing round-the-clock care. It often involves mothers setting aside their personal interests and needs for the sake of their children. In traditional Chinese culture, the adage “the elder sister is like the mother” reflects a similar sentiment (Douglas and Michaels, 2005). As a secondary maternal figure, the elder sister may also find herself ensnared in the complexities of intensive motherhood and facing similar pressures and expectations as those traditionally imposed on mothers.

In today's society, mothers fulfill a multitude of social roles that extend far beyond child-rearing and household management. Motherhood transcends the mere biological aspects of pregnancy and childbirth to encompass a broad spectrum of responsibilities in nurturing and guiding children's development. In family and educational settings, mothers often emerge as the primary caretakers and key participants (Douglas and Michaels, 2005). Consequently, the concept of intensive motherhood goes beyond the mere allocation of time to children's daily care. It also encapsulates a deepening commitment to the educational and overall developmental needs of children. This expanded view of motherhood includes a proactive involvement in their schooling and learning, underscoring the multifaceted nature of modern maternal responsibilities. This broader interpretation of motherhood reflects the evolving societal expectations placed on mothers and highlights their critical role in both the domestic and educational realms.

Jin and Yang (2015) offered a focused discussion on the concept of “education for mothers,” thus highlighting the expanding scope of maternal involvement in education. This involvement spans from the early educational stages of their children to critical milestones like college entrance examinations, with mothers playing a pivotal role at every juncture. Building upon this idea, Yang (2018) introduced the term “brokerization” to describe the intensifying educational responsibilities shouldered by mothers. In this context, children are viewed as high-value projects in the educational sector, with mothers acting as their “educational agents”. This role involves adeptly navigating information networks, understanding the landscape of educational products and the requirements of target schools, crafting personalized learning strategies for their children, and meticulously orchestrating educational resources. This form of intensive mothering represents an evolved version of traditional motherhood that imposes heightened standards and responsibilities. It underscores the increasing pressures faced by mothers in contemporary China, who are expected to go above and beyond to ensure the educational success of their children. This trend reflects a significant change in the perception and expectations of motherhood, particularly in the realm of education.

Peng (2018) observed that despite the increased economic contributions of Chinese women to their families, their traditional gender role as the primary caregiver remains largely unaltered. This situation presents women with inherent conflicts between their professional and family responsibilities, as well as between their personal ambitions and their children's needs. However, Peng emphasized that women are not entirely passive in parenthood. They possess the agency to challenge the constraints of intensive motherhood and mitigate the so-called “motherhood penalty”. By striving for a balance between career and family life, women can assert their subjectivity and initiative. This perspective encourages a reevaluation of maternal roles by advocating for women's empowerment to navigate and reconcile the complexities of work and family dynamics, thereby allowing them to fulfill both their personal and parental aspirations effectively.

Nevertheless, intensive motherhood is challenged by mothers due to different socioeconomic, ethnic, and educational backgrounds. Low-income and women of color and frequently encounter and interpret motherhood in distinct ways compared to their white, privileged-status counterparts. Historically, women of color have had to engage in paid work to provide for their families. As a result, they tend to have a more inclusive perspective on motherhood and paid employment. Collins explains that unlike the conventional notion of the traditional family, which sees paid work as conflicting with and unsuitable for motherhood, Black women consider work as an essential and esteemed aspect of being a mother. Collins et al. delved into the prevalent mothering practices in African American communities, thus challenging the conventional wisdom of entrusting child-rearing solely to one individual. Their research highlights a communal approach to parenting, where other family members or community figures contribute to what they term “team collective mothering” (Wiedmer and Hardy, 2016; Collins, 2022). This model contests traditional gender ideologies that position mothers as the best and primary caregivers for children. Christopher (2012) introduced the concept of “extensive motherhood” and contrasted it to “intensive motherhood”. They entrust a significant portion of the daily child care to others and redefine effective mothering as being in a position of authority and ultimately accountable for their children's welfare. This perspective recognizes that mothers are responsible for childcare and building better material conditions for their children's development. Christopher strategically explored the potential of motherhood to balance the needs of both the mother and the child within professional practices. This includes managing ways to compensate for a mother's partial or complete absence in child rearing, thereby offering a more flexible and inclusive understanding of maternal responsibilities. This approach underscores the possibility of a harmonious balance between maternal roles and other aspects of a woman's life, thus advocating for a broader and more adaptable view of motherhood. Tao offered a distinct perspective from the concept of loosening motherhood (Tao, 2013). She found that urban women are redefining motherhood by treating it as a collaborative endeavor. This approach involves the participation of fathers or other family members to strike a balance and achieve consistency between maternal duties and workplace responsibilities. Such a practice of motherhood, which incorporates the contributions of various family members, can be seen as a mothering model with distinct Chinese characteristics. Qin and Liang (2022) engaged in conversations with mothers of cross-border school children. Their observations revealed that these mothers adapt their cognition and emotions, actively seek surrogate motherhood, and strive to integrate motherhood with livelihood. They employed strategies such as compressing space to gain time to navigate challenges and cultivate a positive mothering experience. Furthermore, prior to China's reform and opening up, China already had the highest number of working mothers in the world. During the socialist period until the late 1970s, the employment rate of women in China was among the highest globally, surpassing 90% (Lavely et al., 1990). Women were expected to attain economic self-sufficiency upon marriage and to actively contribute to the responsibilities of child-rearing. Despite the shifting labor market conditions, women's perception of financial autonomy remains robust. Juggling between work and family necessitate delegation of a portion of caretaking responsibilities to others. Indeed, exclusive mothering practices including Bowlby's theory of attachment flourished much of western literature and industrialized nations with an expectation of normative models of stay-at-home mothers taking care of child within the private sphere (Bowlby, 1969; Seymour, 2013).

Apart from maternal care, omnipresent non-maternal care is displayed and mothers utilize the kinship network to effectively care for their children. Though situations vary from culture to culture, support and caregiving received from older siblings, grandparents and extended kin exist across ethnographic contexts. Konner proved the multiple caregiving practices persisted after hunting-gathering era and exist across cultures. For example, Alloparenting was demonstrated to prove surviorship and nutritional benefits, and it is shown to be beneficial to Hadza (Konner, 2011). The availability of alloparental caregivers include fathers, grandparents and distant relatives (Crittenden and Marlowe, 2008). Hrdy's book Mothers and Others: The Evolutionary Origins of Mutual Understanding believed cooperative breeding is significant in humans evolution and mothers were dependent on grandmothers to raise a child (Hrdy, 2001). Additionally, The Aka presented an important case that allomaternal care is ubiquitous (Meehan and Hawks, 2013). Seymour (2013) investigated all Bhubaneswar households and found that mothers received help from mothers-in-law. Among alloparenting, Seymour argued sibling caretaking was widespread cross-culturally (Weisner et al., 1977). In non-western contexts, caretakers range from extended kin to siblings. In Pastoral and agrarian societies, the most frequent reported caretaking practices and responsibilities were relied upon siblings. For example, the Gusii siblings assumed tremendous caretaking and sooth infants when mothers are absent (Munroe and Munroe, 1980). Roche and Noble-Carr (2017) suggested birth children were virtually responsible for foster children, supporting them emotionally, protecting them from being bullied and responding to their need. Nordenfors (2016) indicated birth children was best described as caregivers on daily basis and completed large amounts of caring tasks.

While alloparenting and sibling caregiving are prevalent across human societies, there are particular conflicts and new features in the context of sister motherhood in contemporary China. At the family level, during the era of the one-child policy, families usually had only one child, which kept parenting costs relatively low, and parenting concepts were more focused and refined. Additionally, influenced by modern cultural patterns, families pursued scientific parenting and orderly living conditions. Nowadays, with the opening up of the two-child and three-child policies, multi-child families are increasing, and parenting costs have risen significantly. Although the concept of scientific parenting still exists, families face higher economic and time investments when raising multiple children, leading to greater parenting stress. Furthermore, in the past, mothers were usually the direct caregivers, focusing on their children's daily lives and education. However, the work pressure in modern society, such as the 996 and 007 work models, has shifted the mother's role from being a direct caregiver to being a family manager, relying more on the help of other family members.

For elder sisters themselves, influenced by the patriarchal system and traditional gender roles, women are expected to take on more caregiving and parenting responsibilities. Older sisters in multi-child families often have to juggle their studies and take care of their younger siblings, highlighting gender tensions. Moreover, as elder sisters transition from being cared for to providing care, they face significant emotional and psychological pressure, needing to balance their own needs with family responsibilities. Simultaneously, society's traditional expectations of women add to their burden.

In summary, the phenomenon of “sister motherhood” in Chinese multi-child families exhibits notable characteristics of the times and unique changes in the cultural background, which deserve further investigation.

### 2.3 Research question

In the historical context of China's policy change to allow the birth of second and third children, many families have expanded to include multiple children. Data from the “Statistical Bulletin on the Development of China's Health Careers,” published by the National Health Commission, indicates a significant trend in this regard. Since 2012, China's Statistical Yearbook^1^ has expanded its coverage to include insights into the reproductive status of women of childbearing age. According to reliable statistics, from 2011 to 2016, second and third births in China constituted a significant share, ranging from 30% to 45%, of the total births in each respective year. More specifically, the 2012 edition of the yearbook reveals that in 2011, the total number of births stood at 9,684, among which the number of second and third children totaled 3,256, accounting for 33.6% of the overall births. Furthermore, the 2013 edition indicates that the proportion of second and third births had risen to 35.6%. These figures underscore the trend of increasing multiple births in China, reflecting societal changes and the evolving fertility policies in the country. From 2017 to 2020, the proportion of second-born children in China stabilized between 50 and 60%. In 2021, out of the 10.62 million births in the country, 41.4% were second children, and 14.5% were third children or higher.^2^ In families with multiple children, it is becoming increasingly common for older sisters to undertake maternal roles by often stepping in for their mothers. This change places the responsibility of motherhood on older sisters, which can offer new and diverse perspectives on motherhood. While extensive research on motherhood has been conducted, both internationally and domestically, the focus has predominantly been on mothers who have experienced pregnancy. In 2021, Chinese scholars broadened the scope of motherhood research to include “mom fans” within fan communities, viewing them as a novel manifestation and bearers of motherhood responsibilities (Xu and Meng, 2021). However, the exploration of sisterly motherhood remains relatively unexplored, and it represents a significant gap in the current understanding of the multifaceted nature of motherhood.

Therefore, this paper aims to explore the initiative and passivity of younger generation sisters engaging with maternal practices by conducting in-depth interviews with 10 sisters around the age of 20. This study builds on existing research on motherhood to identify the characteristics of sisterly maternal roles and summarize the impact that fulfilling these maternal duties has on sisters.

## 3 Research design

This study employs in-depth interviews as its primary research method. Building upon a comprehensive review of literature in the field of motherhood research and integrating insights from previous studies, we conducted semi-structured, in-depth interviews with 10 sisters in their early twenties. These interviews aimed to explore their reasons for assuming maternal roles and their attitudes toward these responsibilities. We aimed to analyze both the proactive and passive aspects of their engagement in motherhood.

### 3.1 In-depth interview

The in-depth interview method is a prevalent technique in qualitative research. It typically involves conducting semi-structured interviews with a set of pre-prepared questions, which allow for a deep exploration of the subject matter. This approach typically garners detailed and profound insights through extended face-to-face conversations with interviewees. The aim is to uncover the interviewee's underlying motivations, beliefs, attitudes, and emotions regarding a particular issue (Yang and Sun, 2005).

One of the primary strengths of in-depth interviews is that they provide interviewees with the freedom to express their thoughts and opinions openly and without constraints. Participants are encouraged to share their experiences and feelings comprehensively, while researchers employ strategic questioning and probing to gain a more profound understanding of their perspectives and thought processes. Furthermore, in-depth interviews enable researchers to uncover the motivations and reasons behind participants' sentiments and experiences.

### 3.2 Research participants

This research targets women who have experienced a form of motherhood. The criteria for participation include having younger siblings with an age gap of 7–20 years and playing a significant maternal role within their families. Utilizing the snowball sampling technique, the study identified 18 potential participants residing in Guangdong Province. These candidates were found through various channels, including social networks, online forums, and family social circles. Following initial outreach and communication, 10 individuals were ultimately selected as research samples. These participants voluntarily agreed to participate in the interviews, providing a diverse and representative sample for the study. This methodical approach ensures a focused and relevant examination of the sisterly maternal experience within the specified demographic and geographic parameters.

Table 1 presents the basic profiles of all the interviewees involved in this study. The group consisted of 10 individuals, whose ages ranged from 20 to 35 years. Most participants hold at least a bachelor's degree, with one participant having a college diploma. Among the interviewees, two are employed and have children of their own, whereas the remaining eight are currently students. Notably, one or both parents of most interviewees are often preoccupied with their jobs. This aspect is relevant, as it influences familial dynamics. The interviewees have younger siblings who are between 7 and 20 years younger than themselves. These participants have witnessed the birth and development of their younger siblings and have been actively involved in their care. They perceive themselves as akin to second mothers to their siblings, which aligns with the concept of “sister-mother” roles. This setup provides a compelling context to explore the dynamics and experiences of sisterly maternal roles.

**Table 1:** Basic personal and family information of the interviewee.

**No**.	**Age**	**Education level**	**Occupation**	**Situation of younger siblings**	**Age gap**	**Family status^a^**
A1	21	Bachelor	Student	One younger sister	9	Mother: anonymous, **
						Father: anonymous, **
A2	22	Bachelor	Student	One younger sister	17	Mother: State-owned enterprise staff, ***
						Father: State-owned enterprise staff, ***
A3	21	Bachelor	Student	One younger brother	9	Father: Decorator, ****
						Mother: Kindergarten teacher, *
A4	30	PhD	University teacher	One younger brother	20	Father: Civil service, ****
						Mother: Teacher, ****
A5	20	Bachelor	Student	One younger brother	7	Father: Service sector, ****
						Mother: Service sector, ****
A6	20	Bachelor	Student	One younger sister	14	Father: Civil service, *
						Mother: self-employed household, ****
A7	20	Bachelor	Student	One younger brother	13	Father: Police, ****
						Mother: Kindergarten teacher, ****
A8	20	Bachelor	Student	One younger brother	13	Father: Lecturer, ****
						Mother: Hotel manager, *
A9	35	College	Salesman	One younger brother	13	Father: Worker, ****
						Mother: Baker, ****
A10	22	Bachelor	Student	Two younger brothers	12	Father: self-employed household, ****
						Mother: self-employed household, ****

^a^The more *, the more busy the people are.

### 3.3 Research procedures

The in-depth interviews for this study were scheduled to be conducted between March and April 2023, with each session lasting range from 30 min to 1.5 h. Prior to the interviews, a set of open-ended questions and potential follow-up queries were meticulously prepared. The interview process and methodologies were thoroughly explained to the participants to ensure their informed consent.

All interviewees consented to audio recording and agreed to provide additional information if necessary. The use of the interview data was approved by all participants for the analysis in this study. Given that some of the questions delve into areas of personal privacy and sensitive information, interviewees were given the option to refrain from answering such questions. It is noteworthy to mention that 1–2 of the interviewees are in special circumstances and are unwilling to answer, or the interviewees are not rich enough to experience, and the interview process is relatively simple. Nevertheless, the majority of participants responded to all the questions. This approach ensures both the ethical handling of sensitive data and the comprehensive collection of relevant information for the research.

## 4 Analysis

### 4.1 Passivity: caused by biological age gap and lack of parental role

Passivity in this context stems from the inherent biological age differences between the older sister and her younger siblings, coupled with the absence or limited presence of a parental figure. This dynamic often leads the elder sister to assume maternal responsibilities more out of necessity than choice, reflecting a passive adaptation to family circumstances. In this study, a significant age gap was observed between the interviewed sisters and their younger siblings. By the time these younger siblings were born, the older sisters had already reached a level of maturity and independence, which equipped them with the ability to care for themselves and others. This maturity led the interviewed women to assume the responsibility of caring for their younger siblings.

Several interviewees expressed this sentiment: “I usually take care of his daily needs because of the age gap.” (A2) and “After all, there is an age gap, so I actually have responsibilities and obligations” (A6). Another pointedly noted, “It's because of the age gap between my brother and me. If I were only one, two, or three years older than my brother, then I would be a child myself, so how could I take care of him?” (A9). This biological factor, the age gap, is immutable, leading the older sisters to accept their caregiving role more passively. Their involvement in their younger siblings' lives is not so much a choice but a circumstance born out of family dynamics and the necessity that arises from these age disparities.

Parentification describes a change in family roles, where children, typically the older ones, assume responsibilities and roles that are conventionally meant for parents. This includes caring for, supporting, and protecting other family members. Parentification is generally categorized into two types: instrumental parentification and emotional parentification. Instrumental parentification involves children being compelled to take on practical familial duties. These responsibilities can range from performing household chores, looking after younger siblings, caring for ill family members, to managing family finances. Essentially, it encompasses tasks that are typically part of a parent's role in the household's day-to-day management. Emotional parentification, on the other hand, involves children shouldering the emotional needs and responsibilities of the family. This may include offering support, comfort, listening, caring, and protection. In some cases, children in this role may act like psychological counselors by assisting family members in dealing with their emotional issues. This form of parentification requires the child to provide emotional support and stability within the family, often at the expense of their own emotional development (Friedman, 1985).

In the families of the sisters interviewed for this study, it was common for the parents to be simultaneously occupied with work and managing the upbringing of two children and often caring for four elderly family members. This scenario placed high work demands and substantial familial responsibilities on the parents, leading to limited energy and time available for family engagement. Additionally, as parents age, their ability to provide the same level of detailed attention and guidance to the upbringing and education of a second child may diminish, possibly resulting in insufficient support for the child's behavior and growth. Consequently, the elder sister often steps into a “parentified” role, taking on extensive family responsibilities and maternal duties, owing to her age and position within the family. This dynamic is reflected in statements from the interviewees: “My parents don't take care of her like they did me before.” (A1) and “I have put a lot of energy into it. The second child is a boy and he is very naughty. The parents are getting older, so they don't have the energy to take care of him anymore.” (A3). According to A4, due to her parents' unfamiliarity with electronic products and their busy work, she took on the responsibility of taking care of her younger brother's daily life and studies during the epidemic. “I get up at 6:30 a.m. every morning to prepare breakfast, and at 7:15 a.m., my brother enters the online morning exercise session, and then starts the day's online classes. And I'm going to be with him the whole time.”

This phenomenon highlights the impact of parental workloads and the challenges of intergenerational caregiving on the family structure, often necessitating older siblings to fill in the gaps of parental care and support. Furthermore, in low-income families where parents work outside the home to augment family income, children are often left unattended. This situation amplifies the responsibility of the elder sister to care for her younger siblings. The burden of childcare and household management frequently falls on these older siblings, who must balance these duties alongside their own needs and responsibilities.

Younger siblings tend to rely heavily on their older siblings for care and guidance. The absence of one parent often necessitates the elder sister to step into a quasi-parental role, providing not only basic care but also discipline and emotional support. This scenario places additional responsibilities on the elder sisters, who become crucial figures in the upbringing and wellbeing of their younger siblings, often at the cost of their personal development and leisure. Due to the demanding nature of work and substantial family obligations, parents often find themselves unable to provide adequate care for their younger children. “During the winter and summer vacations, I am responsible for my younger brothers' three meals a day, school pick-up and drop-off, and after-school tutoring. Sometimes I often wish I could graduate soon”. This situation leads to older sisters involuntarily assuming maternal roles that extend well beyond the typical responsibilities of an elder sibling, which effectively subjects them to parentification. Notably, not all instances of older sisters taking on maternal roles are purely out of necessity. In some cases, these elder siblings are motivated by a sense of love and responsibility, choosing to embrace these duties voluntarily. Their decision to undertake maternal roles is driven by a deep commitment to the welfare of their younger siblings, showcasing a blend of affection and a sense of duty in their family dynamics.

### 4.2 Initiative: driven by love and responsibility

The education received by the elder siblings interviewed, the love they experienced within their families, and their internalization of social values have shaped their expectations and requirements of their younger siblings, which are framed within the context of traditional motherhood. This perspective is often characterized by a desire to see their younger siblings succeed and flourish, akin to the mentality of “women becoming phoenixes”, projected onto their younger brothers and sisters. These elder siblings typically hold aspirations for their younger siblings to conform to societal norms, which emphasizes the importance of good familial upbringing and moral character. This emotional bond encompasses a sense of familial responsibility and care for their younger siblings and mirrors typical maternal traits. Their sentiments are encapsulated in statements like “I have high expectations for my younger siblings…”. “I hope to bring some of the good things I have learned to her” (A2) and “I want to pass on the essence of some things from my education to my brother… It's not so much a conscious effort, as it is an instinctive desire to see my brother become better” (A7). Another interviewee remarked “After all, we share a blood relation… I hope he receives an education more suited to the new generation of young people, within the realm of my understanding and enjoys more on experiential growth.” (A8).

### 4.3 Characteristics of sister-motherhood

#### 4.3.1 All-round and regardless of time

The maternal responsibilities undertaken by the elder sisters interviewed were comprehensive and encompassed various aspects of their younger siblings' daily lives. These duties include, but are not limited to, managing their meals, sleep, and hygiene, as well as overseeing their education, studies, and physical and mental health. The depth of these responsibilities was evident in their descriptions: “When he was just born, his health wasn't great… I had to juggle my studies and also keep track of his bowel movements every day. I would record the time every time he pooped”. (A3). Another shared, “For example, I have to manage his diet. I'm responsible for what he eats, ensuring he doesn't go hungry. I make sure he does his homework and gets to bed on time”. (A5). A third interviewee recounted, “When they were younger, I prepared their milk, changed their diapers, and played with them. Now, I take them to and from school, assist them with homework, and cook their meals” (A10). These elder sisters also expressed a sense of frustration with the intense level of care required: “Guiding them, teaching them experiences—that's fine. It's more about guidance rather than acting like a nanny, hovering over them all day, chasing after them to feed or dress them.” (A3). Another reflected, “Every 24 hours of my vacations belonged to them, and not a minute was my own. I feel like a nanny, always around them, attending to every big or small need. I barely have any time or space for myself.” (A10).

When certain elder sisters engage in caring for their younger siblings, their involvement doesn't necessarily encompass all facets of housework and daily life. In some cases, their role is primarily to accompany their younger siblings in play. This aspect of companionship is often underestimated, with many perceiving it as an effortless and casual endeavor. However, providing companionship is in fact a substantial commitment, demanding considerable time and energy. It constitutes an important form of emotional labor (Pin and Lin, 2020). Motherhood is manifested not only in tangible tasks and caregiving but also in providing emotional support and nurturing. Therefore, in the act of accompanying their younger siblings, elder sisters effectively shoulder a portion of maternal duties. This role encompasses more than mere physical presence; it involves engagement, understanding, and emotional connection, all of which are integral components of motherhood. In this way, the seemingly simple act of playing with and being there for younger siblings carries with it the weight and significance of maternal care and emotional support.

#### 4.3.2 Auxiliary role rather than complete replacement

During the interviews, some elder sisters mentioned that their families had hired a nanny to undertake a portion of the mothering responsibilities. This inclusion of a nanny helped ease the physical demands associated with childcare, thereby alleviating some of the burden on the elder siblings. However, the interviewees also unanimously agreed that while nannies can ensure the children's daily needs and general wellbeing, they cannot fully substitute the emotional support and care that a mother, or in their case, a sister, provides. The sentiment shared by the sisters was clear: “After all, nannies aren't related by blood… They play a supportive role, helping with tasks like making milk or changing diapers. But the provision of time, companionship, and emotional sustenance still needs to come from parents or us, the family members.” (A2). Another sister elaborated, “Being a mother or a family member is not just about taking care of basic needs like food and clothing, but also involves addressing practical problems. It's not enough to just ensure they're fed and clothed, attention must also be paid to their health, education, and other areas. What a nanny can replace is the care related to basic needs, but they cannot provide the emotional support needed.” (A4).

These reflections highlight the complex nature of motherhood and sisterhood roles, underscoring the fact that while some aspects of caregiving can be delegated, the emotional and nurturing components inherent to these roles are irreplaceable and deeply personal. “A nanny cannot fully replace a mother. Although she can provide companionship and care during her time with the child, she can't be there for every aspect of the child's growth.” (A5), encapsulates the sentiment expressed by many interviewees. Another added, “Having the means to hire a nanny can be a great help. It's beneficial in reducing the burden on both my sister and my parents. It's not a replacement but rather a supportive supplement.” (A7). These statements underline the belief that while nannies can alleviate some caregiving responsibilities, they cannot entirely substitute the unique role of a mother or an elder sister. The effectiveness of hired caregiving also came under scrutiny. Interviewee A2, who had experience with employing a nanny, shared, “Finding a good nanny is really challenging. Once they find loopholes in your home, they start to slack off… This resulted in my sister picking up bad habits. Part of the reason we stopped hiring a nanny was due to the negative impact.” (A2). Similarly, A3 observed, “The nanny was only there to take care of his daily needs. He might develop bad habits and become too dependent on others for tasks he should be doing himself.” (A3). Their perspectives are encapsulated in statements like, “If you're not a mother, you'll never fully grasp that feeling. Some aspects are only truly understood through the experiences of pregnancy and childbirth. As a sister, I can only take on part of it, but I can't completely replace a mother.” (A6). Another interviewee reflected, “A sister can never fully take on a mother's responsibilities. The sense of dependency that comes with a mother's role is something a sister can't replicate.” (A8).

### 4.4 Challenges and rewards of sister-motherhood

#### 4.4.1 Challenge: unresolvable physical and mental pressure and emotional burden

The elder sisters interviewed for this study often find themselves juggling their own studies, careers, and in some cases, their families, while also dedicating considerable time and emotional energy to the care of their younger siblings. This often infringes upon their personal rest time, leading to both physical and mental exhaustion. One sister shared her experience: “I still remember being asked by my mother to wake up in the middle of the night to reconstitute formula for my brother. I was so irritable… Everyone else was asleep, and there I was, having to get up and do it. It was really frustrating” (A9). Another interviewee, A4, who has a child of her own, recounted the challenges of simultaneously caring for her child and younger brother. “When my brother was young, and my own child was still breastfeeding, looking after both of them was really tough…. I even had to take them both with me to school and work.” (A4). Interviewee A7 experienced conflicts between her educational commitments and her role in caring for her younger brother. With no one else at home to look after him, especially at an age when he could not be left alone, she often had to bring him along to her tutoring sessions. “In most cases, I compromise. I would rather put myself at a disadvantage than him,” she (A7) explained. Interviewee A10 faces an extraordinary challenge, as she is responsible for her twin younger brothers, effectively doubling her maternal duties. In caring for them, she often feels as though she has lost her own identity, becoming entirely the nanny and mother to her brothers. This intense immersion in motherhood has proven to be overwhelming, taking a significant emotional toll on her. She shared a poignant moment of realization: “My younger brothers were being noisy, and there I was, disciplining them and cooking for them. Their homework still was not done, and I knew I'd have to help them with it after dinner. Suddenly, I just felt so exhausted and incredibly sad. The tears just started flowing out of nowhere” (A10). This account reflects the profound impact that such an intensive caregiving role can have, not only on the physical but also on the emotional wellbeing of an individual. A10's experience underscores the often unseen sacrifices and challenges faced by those who step into substantial caregiving roles within their families.

Women, in placing high expectations on their children's educational achievements, the development of various soft skills, and their physical and mental wellbeing, also place elevated demands on their own personal qualities, such as emotional investment and parenting wisdom (Chen and Luo, 2022). This heightened expectation increases the mental load that women carry within the family. For the elder sisters interviewed, these expectations extend to the future outcomes of their siblings. While fulfilling the maternal role, they also grapple with the uncertainties surrounding their siblings' growth. Any improper actions or behaviors, the development of bad habits, or struggles with academic performance among their younger siblings can trigger self-blame and anxiety. The harsh reality of not having enough time and energy to devote to their younger siblings compounds their feelings of powerlessness. One sister candidly expressed her sense of pressure and helplessness: “I feel an immense amount of pressure. His academic performance is so poor, and our parents aren't able to guide him. What can I do? I feel helpless because neither my parents nor I have enough time to care for him properly, and we fear that he may develop in the wrong direction. My main concern is his overall wellbeing, both physically and mentally” (A4). Another sister voiced her concerns about her younger sibling's excessive screen time: “He spends all day playing on his phone and tablet. I become anxious and deeply worried. What if he develops myopia? What if this affects his future career? I fear that his eyesight might hinder his job prospects” (A7). Interviewee A3's younger brother is in junior high school and likes to use TikTok. Most of the content he pays attention to is about ghosts and animals, and there are also swear words in the videos. A3 is anxious and helpless about this: “TikTok is a mixed bag, with everything on it, which is harmful to his body and mind. It's not very good, and I don't support it. If you don't lead him to the right path now, I, the older sister, will still have to wipe his ass in the future” (A3). Interviewee A10 has not finished her studies and is investing in the education of her two younger brothers. The time in school was actually limited, but as her brother grew up, he lacked discipline and became a bad character with many bad habits, which made her feel guilty and confused. “I'm afraid that I can't teach them well. I think some of my education methods are wrong. But I've never been a parent myself, so I don't know what to do. No one cares about them, and I'm afraid that they will learn poorly in the future. But I feel so powerless and don't know what to do” (A10).

In addition to the pressure from their parents' expectations and their own heightened sense of responsibility, elder sisters often find themselves grappling with the added burden of public opinion and moral judgment from those around them. In the eyes of others, the younger sibling's growth and development have become the older sister's essential duty. This dynamic can lead to misunderstandings and misconceptions, with the elder sister sometimes being unfairly blamed for any perceived shortcomings in her sibling's upbringing. As one sister shared, “Some relatives will comment that your brother isn't studying hard enough, questioning why I, as a teacher, am not actively tutoring him. Some criticize me for not taking my brother under my wing to discipline him, while others suggest that if my mother can't control my brother, I should step in” (A4). Another sister recounted similar experiences, saying, “Some relatives have told me that as the elder sister, it's my responsibility to help him. They wonder who will assist him if I don't. Even some of my brother's teachers have commented that I should pay more attention to his studies” (A10). These statements highlight the societal pressure and expectations placed on elder sisters to play a prominent role in their younger siblings' lives, often resulting in them being unfairly judged or criticized by others when things do not go as expected. Interviewee A8, anticipating the potential pressures and judgments from others regarding her role in her younger brother's upbringing, took a proactive approach. She firmly believed that, given the choice of having a second child, parents should be fully prepared and take appropriate actions. She shared, “Prior to my brother's birth, my parents and I held a family meeting. I made it clear that I didn't want to succumb to societal pressures or hear irresponsible comments from relatives. My parents had discussions with our relatives, so I haven't experienced any such pressures” (A8). On the other hand, the interviewee A4 had a more assertive response to any criticism or judgment directed at her. She emphasized her belief that her parents should be the primary parties responsible for her brother's upbringing and was not willing to accept undue blame. She stated, “I typically don't tolerate this kind of treatment. I don't rely on their family. My parents should be the first ones accountable. Why don't they criticize my dad or mom? Is it because they don't dare to?” (A4).

#### 4.4.2 Return: positive emotional value, growth, and gain

Jane Elstein argued that women's experiences as mothers within the family provide them with a moral advantage when it comes to societal governance (Ueno, 2020). Their involvement in caregiving fosters qualities such as responsibility, empathy, and attentiveness. These virtues, developed through motherhood, can be passed down to the next generation through practice and extended to influence broader social norms and values. This phenomenon is referred to as “social mothering practice.” While the intensive practice of motherhood may pose challenges for most sisters, it is essential to acknowledge that it also offers them positive emotional value and leads to constructive changes. These changes encompass an enhanced sense of personal responsibility and achievement, stronger family bonds, and improved life skills and coping mechanisms. Some statements can support this conclusion. “It made me more patient” (A1). “Because of his arrival, I seem to be more generous, more outgoing, and more willing to socialize and share with others…. In the process of taking care of him, I gradually developed patience and became more attentive” (A7). A4, who already has children, mentioned that as her son grew up, she started to resent her parents' inaction and now feels that “it's beneficial because he (brother) and my son are very close friends, growing up together… Without my brother, I might have been an overprotective mother.” (A4). One of the interviewed sisters also reflected on family relationships through this experience and strengthened the connections and emotional intimacy among family members. “In fact, I now have a better understanding of my parents' hard work, and it has deepened the relationships within our family” (A5). “By observing these relationships—between my brother and my mother, between me and my brother, and between my parents and me, I am better prepared for my own future as a parent and consider the thoughts and preparations I will need to make when I have children someday” (A5).

## 5 Discussion

In ancient China, “sister” was a term used to refer to “mother.” In contemporary China, although the word “sister” has evolved to refer to a woman of the same generation but older than oneself, there are still a few regions where mothers are addressed as “sisters.” While the meaning of the word has changed, the traditional family role of women that it represents has not diminished. Like a mother, the sister plays the role of a “housekeeper” and makes sacrifices for the family. The sisters interviewed experience significant physical and mental pressure due to their motherly roles, and being a mother has become an overwhelming burden. However, no one has seemingly ever asked them whether they are willing to take on this role. Instead, they accept it as a matter of course. “Anyway, if there's nothing to do at home on weekends, I just help take care of my brother… They consider this a very natural thing. After all, I am much older than my brother… In their eyes, I am a grown woman, so isn't it normal for me to help take care of my younger brother?” (A9). Traditional Chinese culture places a strong emphasis on mutual respect among siblings. Older brothers and sisters are expected to take care of their younger siblings, whereas younger siblings should maintain unity and friendship with their elder counterparts. In families with multiple children, parents often have to prioritize work and livelihood, leading older siblings to shoulder the responsibility of looking after their younger brothers and sisters. Parents who grew up in such environments tend to pass down these practices and experiences when raising their own children. My mother used to say: “I had to take care of my younger siblings when I was your age, so you should do the same for your younger brother” (A9). The saying “A sister is like a mother” reflects traditional gender roles and expectations. While it is based on familial love, it has, at times, placed an undue burden on sisters by subjecting them to various injustices. Chizuru Ueno, a Japanese sociologist, has argued in her book “Patriarchy and Capitalism” that attaching symbolic value to “love” and “maternity” and elevating them to a pedestal is an ideology that has historically exploited women's labor (Scott et al., 2008). “This is a tragedy within a patriarchal society, a form of exploitation by both women and society toward their sisters” (A4).

The older sisters interviewed in this study expressed certain contradictions in their approach to caring for and educating their younger siblings. On one hand, they recognize that this excessive caregiving and educational role amounts to a form of moral coercion. They firmly believe that looking after younger siblings should not be an inescapable duty, and they aspire to be relieved from the heavy responsibilities of motherhood. “It's not an obligation. I can assist my sister when I choose to, but I cannot be compelled to fulfill the role of a mother as a sister. I find this unreasonable. If parents decide to have a child, then they should be responsible for taking care of them” (A1). “As a sister, you cannot force me into a mother's role. I believe this is unreasonable. If parents make the choice to have a child, it is their responsibility to care for them.” (A2). On the other hand, driven by a sense of responsibility and the bonds of blood, they find it impossible to turn a blind eye to their younger siblings' needs. Even without external pressure, they willingly take on the role of motherhood. “The societal and traditional cultural expectations in China dictate that you are not just yourself, you belong to someone else. If you are his sister, you must ensure his proper upbringing. Although we acknowledge that we are individuals first, we cannot disregard our duties to our siblings, parents, sons, and daughters” (A4). “After all, he is one of my relatives” (A5). “While there is no necessity or obligation for me to look after him, my conscience would weigh on me. It's almost as if I'm persuading myself” (A6). “It is akin to attending school; it is something that should be done. I don't have to push myself to do it; I naturally take the initiative” (A7). “In any case, I'm a part of this family, and I'll do whatever I can to support the family” (A9).

Furthermore, women who dedicate more time to caring for their children may find themselves particularly susceptible to the influence of early socialization regarding traditional gender roles and family duties. This susceptibility can lead to feelings of overwhelming roles, time constraints, and a sensation of accelerated aging or “maturing faster” (Craig, 2006). One interviewed sister mentioned that assuming the role of a mother had made her “mature earlier, gain more life experience than other girls, and feel more capable of taking care of others” (A9). It's important to note that the concept of “premature puberty” resulting from “sister's motherhood” cannot be seen as a genuine reward. A reward typically refers to positive feedback received as a result of some form of effort rather than the outcome of bearing a burden. This phenomenon actually underscores her self-sacrifice and sense of responsibility. By taking on greater responsibilities and pressure in caring for her younger siblings, she has matured more quickly than her peers. A3's perspective sheds light on an interesting aspect of “sister-in-law motherhood.” She believes that her father's preference for sons over daughters, based on the assumption that she will eventually get married and leave, has made her feel like an outsider in her own family. However, by taking on the role of sister-in-law motherhood, she has experienced an improvement in her family status and has felt a stronger presence within the family. She feels more recognized, responsible, and capable of taking care of others, which has made her more resilient in the face of pressure (A3). However, it's crucial to analyze this recognition critically. While it does affirm her behavior of assuming motherhood, this happens mechanically, without necessarily involving a deep understanding of her feelings and needs. This raises the question of whether this kind of recognition is another form of oppression or neglect. It highlights the complex dynamics at play within families where traditional gender roles are reinforced, and the recognition of women's contributions may not always be based on genuine respect and understanding. Instead, it may be a response to societal norms and expectations rather than an authentic acknowledgment of individual agency and choice.

Furthermore, the experience of “painless motherhood” has imposed significant physical burdens and emotional challenges on the interviewed sisters, burdens that they did not necessarily sign up for. Some have even mentioned that this experience has influenced their future decisions regarding having children of their own. For example, one sister expressed that witnessing the hard work and responsibilities involved in childcare has reduced her desire to have children in the future (A8). Another sister stated that after taking on the responsibilities of motherhood for her younger brother, she is less inclined to have children in the future due to the exhaustion she has experienced (A10).

Taking care of younger siblings has required a considerable investment of time and energy, exposing them to nearly all aspects of motherhood except conception and childbirth. Considering the challenges and demands they have already faced, it is understandable that these younger sisters may hesitate to embrace the prospect of parenthood. The experience of painless motherhood has illuminated the sacrifices and difficulties associated with reproduction, making them more cautious about taking on the costs and responsibilities of raising children.

## 6 Conclusion

The behavior and roles of older sisters are often influenced by traditional family structures and cultural backgrounds. In this study, the interviewed older sisters grappled with the tension between traditional gender role expectations and their individual self-identities. Despite feeling the pressure and challenges associated with assuming the role of motherhood, driven by family affection and moral considerations, they continued to shoulder this responsibility. In society, these sisters often find that their “mother” identity takes precedence over their independent identity. They invest significant time and energy in caregiving but receive limited friendly evaluations and positive feedback from society. These factors collectively contribute to the prevailing negative attitudes and subjective feelings that most of the sisters interviewed harbor toward motherhood. This sentiment is encapsulated by the phrase, “I'm just a sister.” In the meantime, family understanding and support play a crucial role. Without emotional and practical support from their families, the sisters can experience feelings of isolation, helplessness, disappointment, and even anger. Such emotional nourishment is vital for their wellbeing and mental health as they navigate the challenges of taking on the role of motherhood.

In today's society, the trend of having second and third children in quick succession has led to an increasing number of women becoming sisters and taking on the role of mother in China. It is crucial for society to pay attention to the difficulties and pressures faced by these sisters and listen to their inner needs. In the current context of China's imbalanced population structure and declining fertility rates, the distribution of motherhood responsibilities within such families should be reconsidered and reshaped. Creating a warm and supportive family atmosphere for these sisters is vital. Without these changes, they may not consider including childbearing in their life plans. Parents, in particular, need to understand the predicament of their daughters who become sisters and recognize the importance of individual independence. They should understand that the second child is not the responsibility of the first child and should respect their daughter's feelings. This is the key to alleviating their daughters' pressure and dissatisfaction, ultimately leading to improved happiness and stability within the entire family. Furthermore, sisters should not feel obligated to solidify themselves in the role of mother. Instead, they should engage in internal discussions and communication within the family to make the role of motherhood more fluid. This will allow for greater interactivity and flexibility in the role, helping sisters escape the dilemma of internal friction and find a more balanced and fulfilling path forward.

Finally, there are certain limitations to this article. First, the study did not take into account the internal differences between the participants, such as demographic factors such as education, occupation, or marital status, which may have an impact on the results. Secondly, in the course of the interview, out of the protection of privacy and the principle of respecting the interviewee, some interviews may not be in place, which limits the richness of the data and the depth of the research to a certain extent. In addition, the research perspective of this paper mainly focuses on the elder sister herself, and there is a lack of field data from other family members, and the in-depth analysis from the perspective of family structure is also a shortcoming of this study. In future research, we will continue to deepen the interviews and gather more comprehensive and detailed information in order to more accurately reveal the essence of the problem.

## Data Availability

The original contributions presented in the study are included in the article/supplementary material, further inquiries can be directed to the corresponding author.

## Footnotes

^1^ https://www.stats.gov.cn/sj/ndsj/

^2^ http://www.nhc.gov.cn/cms-search/xxgk/getManuscriptXxgk.htm?id=51b55216c2154332a660157abf28b09d

## Appendix

Interview questions (Excerpt)

**Family situation**

1) What is your current age? Profession? Are you married or not?
2) How many younger brothers and sisters do you have? How old are you and your brother/sister?
3) Who primarily takes care of the younger brother (sister) in your family?

**Identity**

1) Why do you feel like a second “mother” to your younger siblings?
2) Do you recognize your identity as a “mother,” and if so, why?
3) Initiative and Passivity
4) What led you to take on the role of “mother”?
5) If there were no external pressure and expectations, would you choose to take the initiative in assuming this “mother” responsibility? Why or why not?

**Challenges and rewards**

1) What are the most significant positive feelings and changes that taking on motherhood has brought you?
2) What troubles and inconveniences does motherhood bring to your life?

**Reflection and discussion**

1) Have your family members instilled in you the concept that “older sisters should take care of younger brothers and sisters”?
2) What is your personal perspective on this concept?
3) Do you believe that the phenomenon of “sisters acting as mothers” is common? How do you evaluate this phenomenon?
